# Supplementary material for: Cement-Based Composites Containing Oxidized Graphene Nanoplatelets: Effects on the Mechanical and Electrical Properties
Source: Nanomaterials (Basel). 2023 Feb 27;13(5):901. doi: 10.3390/nano13050901 (PMC10005776; doi:10.3390/nano13050901)
Supplement: Supplementary file 1 [file nanomaterials-13-00901-s001.zip › nanomaterials-2208480-supplementary.pdf]

Communication

Cement-Based Composites Containing Oxidized Graphene Nanoplatelets: Effects on the Mechanical and Electrical Properties

Luca Lavagna[1,2,*], Andrea Santagati[1], Mattia Bartoli[2,3], Daniel Suarez-Riera[2,4], and Matteo Pavese[1,2,*]

[1] Department of Applied Science and Technology, Politecnico di Torino, C.so Duca degli Abruzzi 24, 10129, Torino, Italy; andrea.santagati@studenti.polito.it

[2] Consorzio Interuniversitario Nazionale per la Scienza e Tecnologia dei Materiali (INSTM), Via G. Giusti 9, Florence, 50121, Italy

[3] Center for Sustainable Future, Italian Institute of Technology, Via Livorno 60, Turin 10144, Italy; mattia.bartoli@iit.it

[4] Department of Structural, Geotechnical and Building Engineering, Politecnico di Torino, C.so Duca degli Abruzzi 24, 10129 Torino, Italy; daniel.suarez@polito.it (D.S-R.)

* Correspondence E-mail(s): luca.lavagna@polito.it; Tel.: (+39)0110904598 (L.L.). matteo.pavese@polito.it; Tel.: (+39)0110904674 (M.P.).

Supporting materials

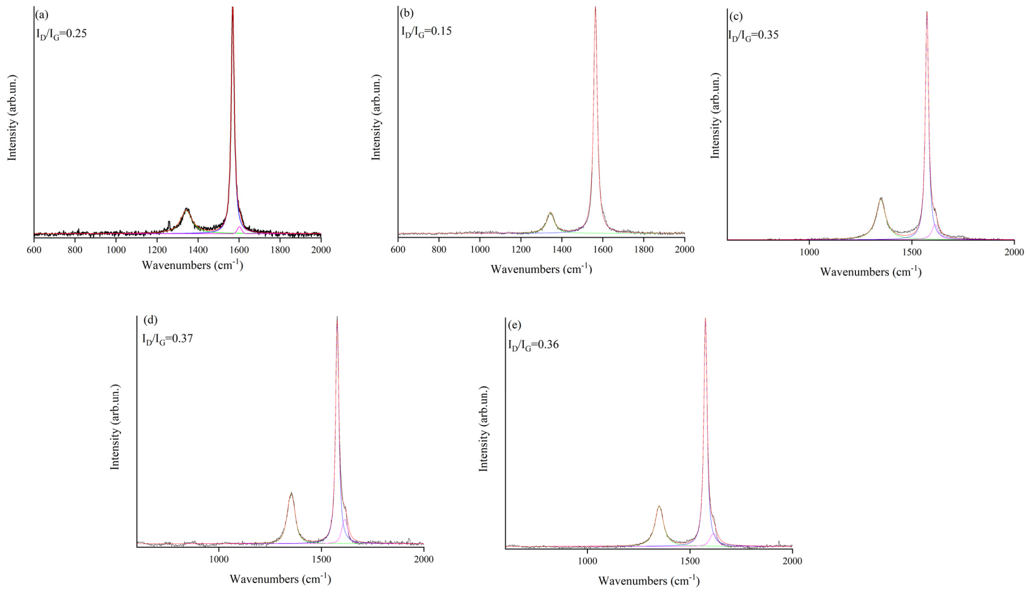

Figure S1. Raman plot of: GNP (**a**), GNP 10′ (**b**), GNP 20′ (**c**), GNP 40′ (**d**), and GNP 60′ (**e**).

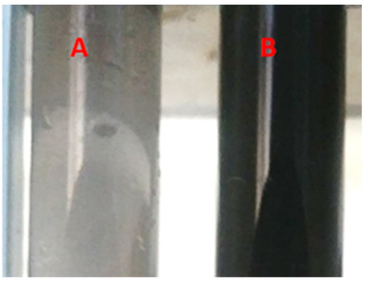

Figure S2. Dispersion of pristine GNP (**A**) and GNPs treated with sulfonitric acids for 60 minutes (**B**) in water.

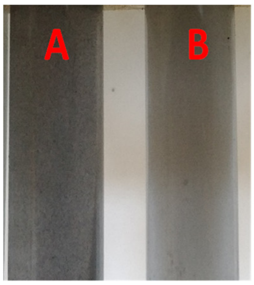

Figure S3. Dispersion of pristine GNP (**A**) and GNPs treated with sulfonitric acids for 60 minutes (**B**) in water after adding 3 ml of calcium hydroxide solution.

Disclaimer/Publisher's Note: The statements, opinions and data contained in all publications are solely those of the individual author(s) and contributor(s) and not of MDPI and/or the editor(s). MDPI and/or the editor(s) disclaim responsibility for any injury to people or property resulting from any ideas, methods, instructions or products referred to in the content.
